# SCFusion: Infrared and Visible Fusion Based on Salient Compensation

**DOI:** 10.3390/e25070985

**Published:** 2023-06-27

**Authors:** Haipeng Liu, Meiyan Ma, Meng Wang, Zhaoyu Chen, Yibo Zhao

**Affiliations:** 1 Faculty of Information Engineering and Automation, Kunming University of Science and Technology, Kunming 650500, China20212204010@stu.kust.edu.cn (Z.C.);; 2 Yunnan Province Key Laboratory of Computer, Kunming University of Science and Technology, Kunming 650500, China

**Keywords:** image fusion, salient compensation, infrared and visible images, deep learning

## Abstract

The aim of infrared and visible image fusion is to integrate the complementary information of the two modalities for high-quality fused images. However, many deep learning fusion algorithms have not considered the characteristics of infrared images in low-light scenes, leading to the problems of weak texture details, low contrast of infrared targets and poor visual perception in the existing methods. Therefore, in this paper, we propose a salient compensation-based fusion method that makes sufficient use of the characteristics of infrared and visible images to generate high-quality fused images under low-light conditions. First, we design a multi-scale edge gradient module (MEGB) in the texture mainstream to adequately extract the texture information of the dual input of infrared and visible images; on the other hand, the salient tributary is pre-trained by salient loss to obtain the saliency map based on the salient dense residual module (SRDB) to extract salient features, which is supplemented in the process of overall network training. We propose the spatial bias module (SBM) to fuse global information with local information. Finally, extensive comparison experiments with existing methods show that our method has significant advantages in describing target features and global scenes, the effectiveness of the proposed module is demonstrated by ablation experiments. In addition, we also verify the facilitation of this paper’s method for high-level vision on a semantic segmentation task.

## 1. Introduction

It is difficult to obtain high quality images during image acquisition due to weather, environment, etc. [1,2]. To improve image quality, researchers have proposed various image processing technology methods [3,4], and image fusion, as an image enhancement technique, can synthesize the complementary information between images to maximize the details of the imaged scene [5]. Among them, infrared and visible image fusion has become a hot research topic in the field of image processing due to its applications in the military and other fields [6]. Visible images usually contain a large number of texture details, but they are susceptible to environmental effects; in contrast, infrared images have the feature of highlighting thermal targets, but infrared images have problems such as a lack of background information, noise, and low resolution [7]. Therefore, the complementary characteristics of infrared and visible images enable their fusion to comprehensively describe the imaging scene, thus providing more feature information for subsequent advanced vision tasks such as pedestrian detection [8], image segmentation [9], etc.

Most of the existing methods for infrared and visible image fusion include some traditional methods and deep learning methods. The traditional methods mainly include multi-scale-decomposition-based methods [10,11], sparse representation-based methods [12,13], subspace-based methods [14], saliency-based methods [15], and hybrid methods [16]. However, most traditional methods achieve image fusion by complex mathematical transformations and hand-designed fusion rules; therefore, they cannot adapt to increasingly complex fusion scenarios. Additionally, due to the powerful deep feature extraction ability of deep learning, it has received more and more attention from scholars in the field of image fusion. Deep learning-based fusion methods are divided into three main categories: auto-encoder(AE)-based methods [17,18,19,20], convolutional neural network (CNN)-based methods [21,22,23,24], and generative adversarial network (GAN)-based methods [25,26,27,28]. 

Although deep-learning-based image fusion methods have been able to generate satisfactory fused images in recent years, there are still some pressing challenges in the field of image fusion. On the one hand, existing fusion algorithms [22,23,27] have a prerequisite: the assumption that infrared images provide salient target information and visible images provide background texture information, which holds under certain conditions (when visible images contain more information), but when conditions such as poor lighting of the visible image imaging scene are poor, this assumption leads to loss of background information in the fused image and the problem of target contrast degradation. On the other hand, there are some self-encoder-based methods [17,18,19,20] that use hand-designed fusion strategies to fuse depth features; however, the depth features tend to be uninterpretable, and the hand-designed fusion strategies are not able to assign appropriate weights to the depth features so that they are not better able to fuse the features. In contrast, some end-to-end methods [22,23,29] use feature fusion by simply cascading the feature information at the end of the feature extraction network, which is susceptible to the loss of shallow detailed texture feature information. In addition, existing methods do not pay attention to the infrared region at the target level when constructing the loss function, which cannot target the saliency of the infrared target in the fused image, resulting in the inevitable weakening of the infrared target in the fused image.

To solve the above problems, we propose a salient-compensation-based fusion framework for infrared, and visible images, called SCFusion. We will describe our approach in detail in Section 3. Overall, our main contributions are four-fold:

- It is presented a saliency-compensated infrared and visible image fusion framework consisting of a multi-scale edge gradient block (MEGB), a salient dense residual module (SRDB), and a spatial bias module (SBM). The fused images have significantly enhanced target information and rich scene descriptions.
- A scene texture mainstream consisting of multi-scale edge gradient blocks (MEGB) is designed to effectively extract the scene texture features of the source image, and the visible and infrared images can complement each other as scene texture information in different scenes, effectively solving the limitation of visible images by low-light scenes.
- A salient tributary trained individually by salient loss is designed, which uses the salient dense residual module (SRDB) to extract saliency targets, improving the target capture capability of the fusion network and eliminating the problem of low contrast in target regions of existing methods.
- A spatial bias module (SBM) is designed to compensate infrared features into texture features at different stages, where information extraction and fusion compensation are performed simultaneously, without the need to design additional fusion strategies.

The remainder of this paper is organized as follows. Section 2 briefly describes the related works of image fusion. In Section 3, we introduce our proposed SCFusion in detail, including network architecture and loss function. Section 4 illustrates the impressive performance of our method in comparison with other alternatives, followed by some concluding remarks in Section 5.

## 2. Related Work

### 2.1. Infrared and Visible Fusion

Deep learning has been sufficiently applied in computer vision tasks including image fusion due to its powerful capability of adaptation, numerous methods based on deep learning have been proposed, which are broadly classified into the following three main categories:

AE-based image fusion: Most of the self-encoder-based methods pre-train on large datasets to obtain encoders and decoders to implement the process of feature extraction and reconstruction, followed by feature fusion using manually designed fusion rules. DenseFuse [17] consists of a convolutional layer, a fusion layer, and a dense block, while the fusion layer is implemented by simple addition and parametrization. To further improve the feature extraction, NestFuse [18] and RFN-Nest [30] introduced nested connections and residual dense blocks in the network. Later, in order to make the network pay attention to specific regions of the source image, Jian et al. [31] employed an attention mechanism to focus on salient targets and texture details of the source image. Xu [20] et al., applied dissociative representation learning to a self-encoder approach considering the interpretability of feature extraction. 

GAN-based image fusion: Generative adversarial networks (GANs) are able to effectively model data distribution even without supervised information, making the network remarkably compatible with infrared and visible image fusion tasks. FusionGAN [25] is the first approach to implement GANs into infrared and visible image fusion tasks, which defines the fusion task as an adversarial game between generators and discriminators. However, with a single discriminator, it is susceptible to a break in the balance of the data distribution between infrared and visible images; therefore, Ma et al., proposed DDcGAN [26], which proposes a dual-discriminator adversarial generative network. AttentionGAN [32] incorporates an attention mechanism based on DDcGAN [26], which intends to have the network retain the target information of infrared images and background information. Additionally, later, Zhou et al. [27] proposed an approach to generate adversarial networks with gradient and intensity discriminators as multi-task fusion, which imported gradient and intensity into the GAN to make the network pay more attention to the gradient and intensity of infrared and visible images.

CNN-based image fusion: Infrared and visible image fusion methods based on convolutional neural networks (CNN) achieve end-to-end feature extraction, fusion, and reconstruction by designing network structures and loss functions. RXDNFuse [33] combines the advantages of DenseNet [17] and ResNet [34] to propose residual dense networks for a more comprehensive extraction of features at different scales. SeAFusion [29] proposed an approach to drive the fusion task with semantic loss to better integrate the fusion task with subsequent advanced vision tasks. Li et al. [35] proposed a dual-attention-based feature fusion module based on the theory of meta-learning, in which the network accepts source image inputs of different resolutions. STDFusionNet [22] proposed the use of target masks to assist in extracting the target of the visible image and the background of the visible image as a way to improve the fusion effect, but the labeling of the mask is manually labeled, which results in a large preliminary workload. PIAFusion [7] considers the lighting conditions, although it embeds the lighting probability into the loss function, which is prone to the problem of overexposure to the background of the daytime scene.

### 2.2. The High-Level Vision Tasks

As one of the important methods in the field of computer vision, semantic segmentation aims to predict the semantic category of each pixel in an image; it has crucial importance in the field of autonomous driving [36]. However, many semantic segmentation methods are designed based on the conditions of good illumination, while the performance of these methods decreases when the image has poor illumination conditions or is occluded. Therefore, it has become a new problem in the field of semantic segmentation to improve the accuracy of segmentation networks when the visible images are contaminated. Some researchers have started to experiment with semantic segmentation methods that combine infrared images with visible images, and most of these methods also involve the process of infrared and visible image fusion. RTFNet [37] employs ResNet to extract the features of two source images separately as an encoder; multimodal fusion is implemented by accumulating the feature blocks of RGB and Thermal encoder paths over the elements, with an upception block designed to recover the feature map resolution. AFNet [38] computes the infrared image and visible image by designing the attention fusion module to the spatial correlation between feature maps while guiding the fusion of features from different modalities in the process. AMFuse [39] was designed specifically for multimodal fusion with an add–multiply fusion block fusing common and complementary features of infrared and visible images, with an attention module and a spatial pyramid pool module added to the module to enhance the information in multi-scale contexts.

However, infrared and visible image fusion methods ignore the variation in complementary information of infrared and visible images in normal light and low-light environments. Therefore, we propose a new fusion method that is able to sufficiently exploit the features of infrared and visible images under different lighting conditions, so as to retain more meaningful information.

## 3. Methods

### 3.1. Network Architecture

**Figure 1:**
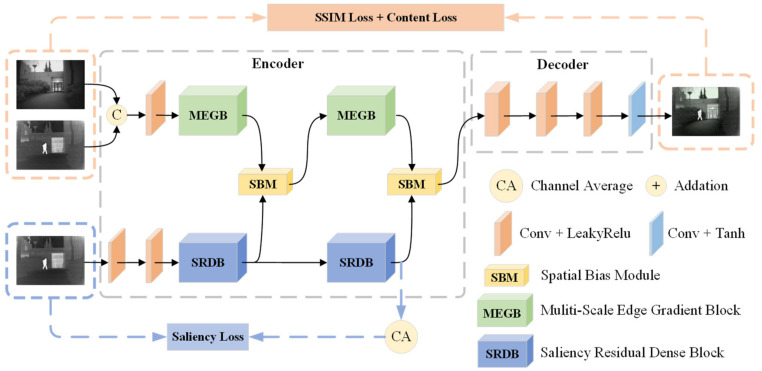
Overall framework for SCFusion. It consists of multiscale edge gradient block (MEGB),salient dense residual block (SRDB), and spatial bias block (SBM). The saliency map generated by the saliency tributary is pre-trained by saliency loss, which is then sent to the main network to generate the fused image with the texture features obtained by MEGB under the joint training of structural similarity loss and content loss.

In order to balance the background texture details of the infrared and visible images without limiting the light conditions of the input image and to enhance the contrast between the infrared target and the scene, we designed the saliency-compensated fusion network, whose overall network is shown in Figure 1. The framework mainly consists of the multiscale edge gradient block (MEGB), the salient dense residual module (SRDB) and the spatial bias module (SBM). The visible and infrared images are integrated into the texture mainstream together to obtain enhanced texture features, while the infrared images are integrated into the salient mainstream to obtain enhanced salient features, both of which are effectively fused with global and local information by the spatial bias module (SBM). The relevant modules will be described in detail below.

#### 3.1.1. Multiscale Edge Gradient Block (MEGB)

**Figure 2:**
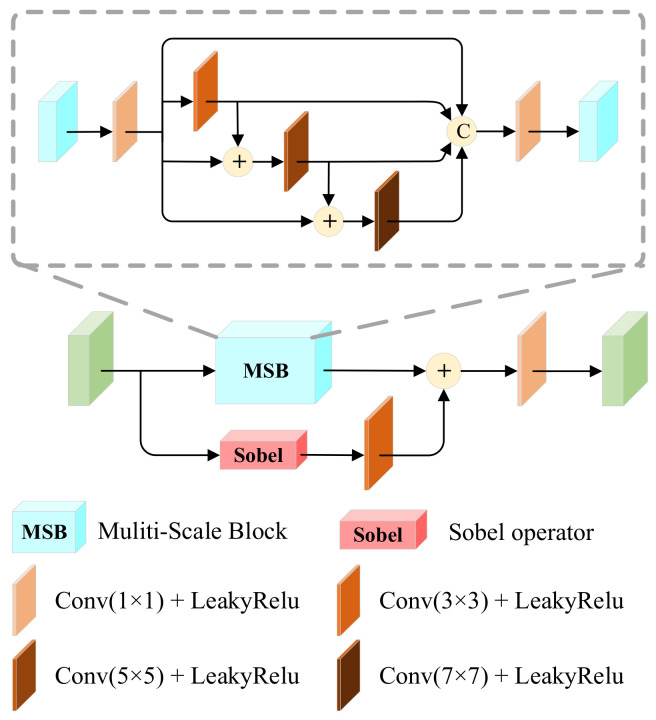
Multiscale edge gradient block (MEGB). It accomplishes texture detail enhancement by combining the output of the multi-scale with the output of the residual gradient flow.

The specific structure is shown in Figure 2, which consists of multiscale mainstream and residual gradient streams. Most networks use convolutional layers of the same size convolutional kernel to extract features, which is difficult to perceive the information comprehensively. So, the multiscale mainstream is added with branches of convolutional layers of different sizes of convolutional kernels to increase the perceptual field. To reduce the information loss in the multi-scale features, different convolutional computations are not added with pooling layers, while the residual gradient flow is combined with the Sobel operator to maintain the strong texture rationality of the features. The multiscale output is then combined with the output of the residual gradient flow to complete the texture detail enhancement.

Specifically, in the feature mainstream, we are given a pair of strictly aligned infrared images $I_{ir}$ and visible images $I_{vi}$, which are approximated by a shallow convolutional layer for modal differences and then joined in the channel dimension to obtain $\Phi_{H}$ In the tributary stream, the infrared images $I_{ir}$ are passed through a shallow convolutional layer to obtain $\Phi_{C}$ $\Phi_{H}$ is directly input to MEGB, and MSB uses different convolutional kernels to extend the perceptual field of the network, and multi-scale features $\Phi_{D}$ cascade to enhance the feature description. The module MSB output feature $\Phi_{M}$ can be expressed as:

(1) $$\Phi_{M}=Conv\left(C\left(\Phi_{D}\right)\right),n\in \left\{1,3,5,7\right\}$$

The texture extraction of the hybrid features is also performed using the Sobel operator to enhance the features’ fine-grained representation, and the above process can be expressed as follows:

(2) $$\Phi_{T_{1}}=Conv\left(Conv\left(\nabla_{Sobel}\Phi_{H}\right)⊕\Phi_{M}\right)$$

where $Conv\left(\cdot \right)$ denotes the convolution operation, $C\left(\cdot \right)$ denotes the cascade on the channel dimension, $\nabla_{Sobel}$ denotes the Sobel operator, and ⊕ denotes element-wise summation.

In summary, MEGB breaks the limitation of texture extraction from lighting conditions by combining multi-scale features and Sobel texture features in parallel to maximize texture details in infrared and visible images.

#### 3.1.2. Salient Dense Residual Block (SRDB)

**Figure 3:**
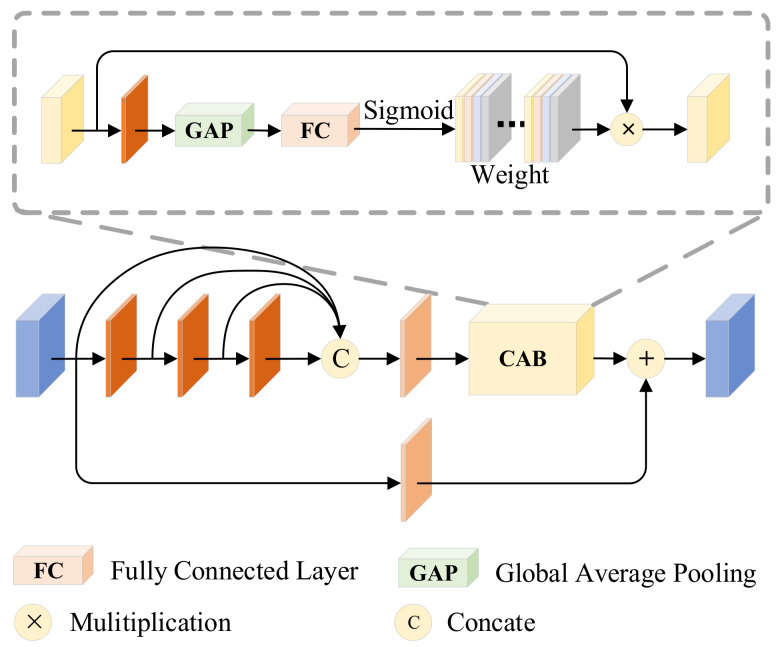
Salient dense residual block (SRDB). It achieves contrast enhancement by combining attentional features with residual flow features.

The specific structure is shown in Figure 3, which integrates dense connectivity [17], residual streams [35], and channel attention (CAB). To obtain comprehensive feature information, we introduce dense connectivity in the mainstream, but to address the high memory cost and energy consumption due to feature reuse, it is replaced by aggregating the features of all previous layers in the last layer of dense connectivity. Densely connected features are input to attention in order to make the network more focused on the attention region. It is remarkable that we generate salient target images in the training phase, while the infrared salient target features are input directly into the subsequent network in the inference phase.

Specifically, we send $\Phi_{C}$ into the SRDB, and after feature reuse, feature $\Phi_{E}$ can be represented as:

(3) $$\Phi_{E}=C\left(\Phi_{C},Conv\left(\Phi_{C}\right),Conv^{2}\left(\Phi_{C}\right),Conv\left(\Phi_{C}\right)\right)$$

The attention first passes through a 3 × 3 convolutional layer, followed by a global average pooling to obtain the global feature vector, a fully connected layer to learn the importance of each channel, and then a sigmoid activation function to obtain the weights and assign higher weights to the features with higher contrast, and multiply the weights with the original input features to obtain the attention feature $V_{C}$.

Finally, the contrast enhancement is achieved by adding the attention features with the residual stream features to highlight the salient targets, and the above process can be defined as:

(4) $$V_{C}=Sigmoid\left(FC\left(GAP\left(Conv\left(\Phi_{E}\right)\right)\right)\right)\cdot \Phi_{E}$$

(5) $$\Phi_{S_{1}}=Conv\left(\Phi_{C}\right)⊕V_{C}$$

where $GAP\left(\cdot \right)$ denotes the global average pooling, $FC\left(\cdot \right)$ denotes the fully connected layer, $Sigmoid\left(\cdot \right)$ denotes the activation function, and $\Phi_{S_{1}}$ is the final output feature of SRDB.

In a nutshell, SRDB calculates the contrast of features on the basis of channel attention to achieve contrast enhancement, which further preserves the high contrast of infrared targets.

#### 3.1.3. Spatial Bias Block (SBM)

**Figure 4:**
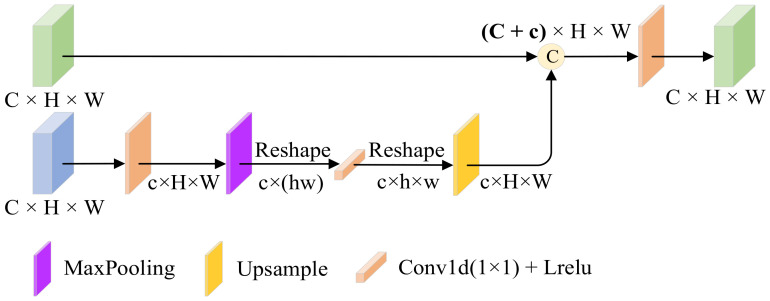
Spatial bias block (SBM). It allows the network to learn both local and global information by connecting spatially biased features with texture features in channel cascades.

The specific structure of the module is shown in Figure 4. The module has two inputs, a texture feature from the mainstream and a salient feature from the tributary. In the salient tributary we focus on the infrared target; meanwhile, we also need to learn the relationship between different distant targets, i.e., the global information to enhance the semantic information of the image, but the simple convolutional layer has the problem of not being able to learn the long-range dependencies due to the limited perceptual field, so we learn the global information by adding a spatial bias channel to the texture tributary. This module is lightweight, unlike the self-attention operation which is too burdensome. The spatial bias term B can be expressed as:

(6) $$B=Relu\left(BN\left(\Phi_{S_{1}},SB\right)\right)$$

where $B\left(\cdot \right)$ denotes the output of the significant features after adding the spatial bias term, SB denotes the spatial bias, and BN and Relu denote the batch normalization and nonlinear activation layers, respectively.

Instead, textures are represented by the grayscale distribution of pixels and their surrounding spatial domains, i.e., local information. By cascading spatial bias features with texture features in the channel direction, the network can learn both local and global information. In order to aggregate global knowledge in the feature map, we use 1 × 1 convolution in the passband dimension. Finally, texture feature $\Phi_{T_{1}}$ is spliced with saliency feature $\Phi_{S_{1}}$ to complete the process of asymptotic fusion, which can be expressed as

(7) $$\Phi_{T_{1}}^{\prime }=Conv\left(C\left(\Phi_{T_{1}},B\left(\Phi_{S_{1}}\right)\right)\right)$$

In conclusion, a simple and efficient fusion rule is the key to image fusion, and SBM utilizes lightweight spatial bias terms to fuse local and global information without increasing the complexity of the network.

### 3.2. Loss Function

We know that under different lighting conditions, image texture information may exist in either visible or infrared images; the salient targets are more prominent in infrared images. Therefore, our method aims to fully extract texture details in both infrared and visible images from the texture mainstream while enhancing the salient targets weakened by the mainstream from the saliency tributaries. Therefore, our method is a two-stage model trained by the mainstream loss function and the tributary loss function, and its training process is shown in Algorithm 1.
 **Algorithm 1:** Training procedure
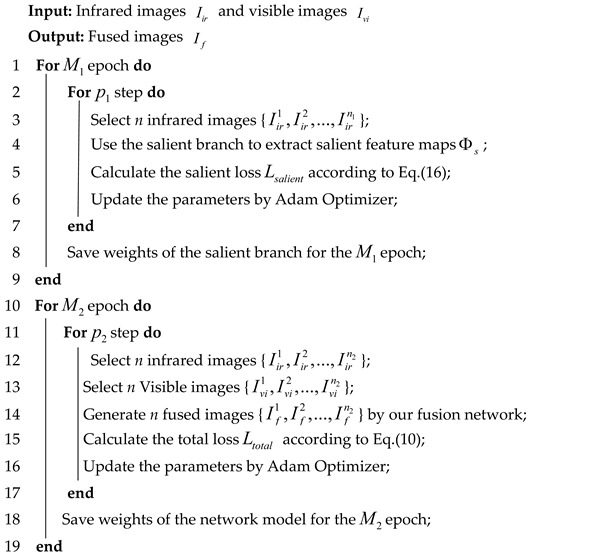


#### 3.2.1. Mainstream Loss

The mainstream branch aims to make the fused image retain rich texture details and improve the visual quality and evaluation index, so we design the structural similarity loss and content loss to guide the network to generate the fused image; the formula of fusion loss is as follows:

(8) $$L_{F}=\lambda_{1}L_{SSIM}+\lambda_{2}L_{Content}$$

where $\lambda_{1}$, $\lambda_{2}$ are the weighting factors to balance the two losses. The two loss functions are described in detail below.

##### Structural Similarity Loss

For the fusion task, we want to close the similarity between the fused image and the source image to improve its fusion performance so that the visual effect of the image is more in line with the visual effect perceived by human eyes. Structural similarity (SSIM) can effectively evaluate the similarity between the source and fused images, which consists of three components: luminance similarity, contrast similarity, and structural similarity. The loss of structural similarity is formulated as follows:

(9) $$L_{SSIM}=1-\frac{SSIM\left(I_{f},I_{VI}\right)+SSIM\left(I_{f}.I_{ir}\right)}{2}$$

(10) $$L_{SSIM}\left(x,y\right)=\frac{\left(2\mu_{x}\mu_{y}+C_{1}\right)\left(2\sigma_{xy}+C_{2}\right)}{\left(\mu_{x}^{2}+\mu_{y}^{2}+C_{1}\right)\left(\sigma_{x}^{2}+\sigma_{y}^{2}+C_{2}\right)}$$

where $I_{f}$ denotes the fused image, $I_{ir}$, and $I_{vi}$ denote the infrared image and visible image, respectively; $SSIM\left(x,y\right)$ indicates the calculation of the structural similarity between two images; $\mu_{x}$ and $\mu_{y}$ are the averages of all pixels in the two source images; $\sigma_{x}$ and $\sigma_{y}$ are the variances of the pixel values of the two source images; and $C_{1}$ and $C_{2}$ are constants to ensure the stability of the function.

##### Content Loss

In addition, our texture mainstream expects the fused image to retain abundant texture details while maintaining the best intensity distribution, so the content loss is introduced, which consists of two parts: intensity loss and texture loss. The content loss is defined as follows:

(11) $$L_{Content}=L_{Int}+\alpha L_{Grad}$$

where $L_{Int}$ denotes the intensity loss,$\;L_{Grad}$ denotes the gradient loss, and ∂ is used to obtain a balance between the strength loss and texture loss.

The intensity loss measures the intensity distribution between the fused image and the source image at the pixel level, so the intensity loss is defined as follows:

(12) $$L_{Int}=\frac{1}{HW}||I_{f}-Max{\left(I_{ir},I_{vi}\right)||}_{1}$$

where *H* and *W* are the height and width of the input image, respectively, and ${||\cdot ||}_{1}$ represents $l_{1}-norm$.

In addition, to encourage clearer texture details, we expect the gradient of the fused image to be close to the gradient maximum of the visible and infrared images, so the texture loss is defined as follows:

(13) $$L_{Grad}=\frac{1}{HW}||\left|\nabla_{Sobel}I_{f}\right|-Max{\left(\left|\nabla_{Sobel}I_{ir}\right|,\left|\nabla_{Sobel}I_{vi}\right|\right)||}_{1}$$

where $\nabla_{Sobel}$ denotes the Sobel gradient operator, which measures the gradient texture of the image; $\left|\cdot \right|$ denotes the absolute operation.

#### 3.2.2. Salient Loss

The purpose of the fusion task is to serve the subsequent advanced vision task, and the salient target is crucial for the subsequent task, so in order to preserve the salient target of the fused image, we use the target mask to construct the intermediate salient loss, which is defined as follows:

(14) $$L_{Salient}=\frac{1}{HW}||I_{m}\cdot I_{ir}-CA{\left(\Phi_{ir}\right)||}_{1}$$

where *I_m_* denotes the target mask, and *CA* denotes the channel average.

In summary, our network of significant target compensation is able to obtain ideal texture details with significant targets guided by structural similarity loss, content loss, and salient loss, and can round-the-clock fuse the meaningful information of source images.

## 4. Experimental Validation

### 4.1. Experimental Configurations

In this paper, we conducted extensive qualitative and quantitative experiments on three datasets, including TNO [40], MSRS [7], and M3FD [28], to comprehensively evaluate our approach and validate the generalization of our method. In addition, we selected seven methods such as DenseFuse [17], RFN-Nest [30], Fusiongan [25], SDNet [41], U2Fusion [23], FLFuse [24], and PIAFusion [7] for comparison with our method. 

The experimental results of visualization are subjective, in this paper, we introduce the standard deviation (SD), visual information fidelity (VIF), and the average gradient (AG). The difference correlation sum of SD is based on statistical concepts to evaluate the distribution and contrast of fused images, and VIF is based on the human visual system designed to measure the fidelity of information from the perspective of human visual perception. SCD measures the correlation between the information of the fused image and the corresponding source image, EN evaluates the amount of information contained in the fused image from an information-theoretic perspective, and SF evaluates the texture details contained in the fused image by calculating the row frequency and column frequency. All the above evaluation metrics are of higher values, indicating better image quality.

This paper presents a two-stage model, so we train the textured main stream and the salient tributary in turn. In the first stage, we train the salient tributaries: epoch = 10. After that, the output features of SRDB are supplemented as mainstream saliency features. Then train the fusion network: epoch = 8. In the training phase of the experiments, a data augmentation method was used to address the problem of small existing visible and infrared image fusion datasets, and a common dataset of aligned visible and infrared images, MSRS was used as the training set. For the hyper-parameter setting: $\lambda_{1}=1$, $\lambda_{2}=15$, α=3. Additionally, we leverage the Adam optimizer with a batch size of 64. The learning rate is $1\times 10^{-4}$. The test set was selected from the public datasets TNO, RoadScene, MSRS and M3FD for infrared and visible image fusion, and 42, 20, 361 and 300 pairs of images each were selected for algorithm comparison experiments. The experiments in this paper were conducted on a GeForce RTX 2080Ti 11GB with PyTorch as the deep learning framework. All comparison algorithms in the experiments were experimented with in the original thesis setup.

### 4.2. Comparison Experiments

#### 4.2.1. Qualitative Results

**Figure 5:**
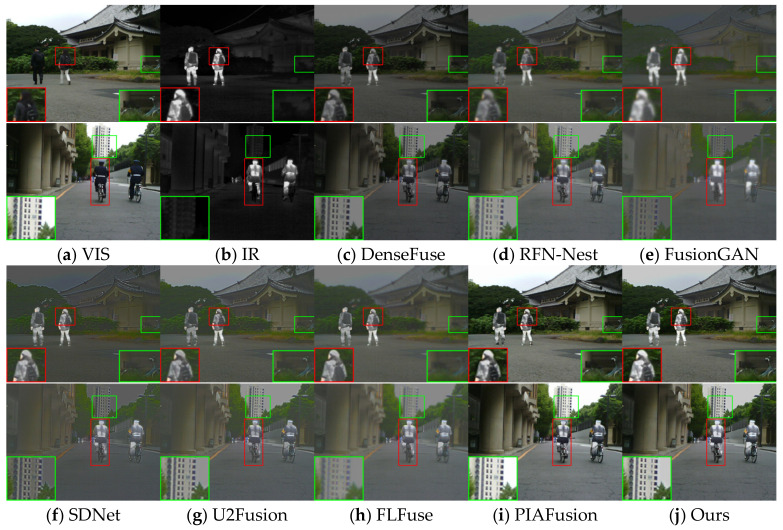
Vision quality comparison on the MSRS dataset. Areas with large differences are highlighted by RED and GREEN boxes, and enlarged images of RED boxes are in the lower right or left corner.

**Figure 6:**
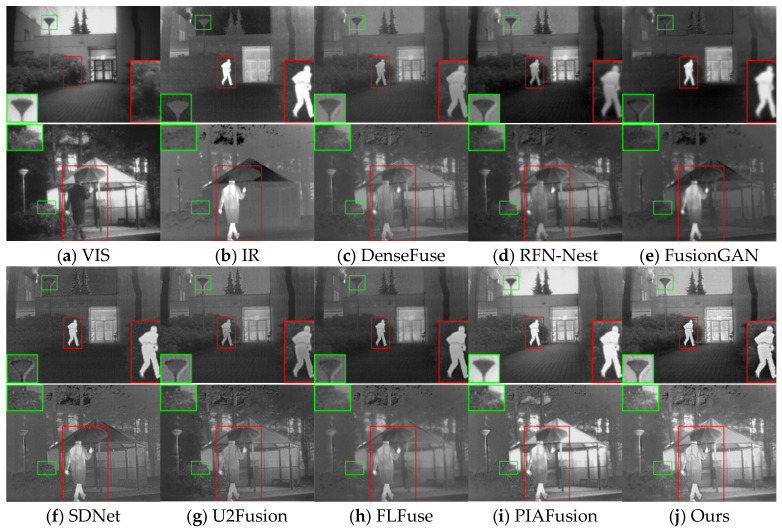
Vision quality comparison on the TNO dataset. Areas with large differences are highlighted by RED and GREEN boxes, and enlarged images of RED boxes are in the lower right or left corner.

**Figure 7:**
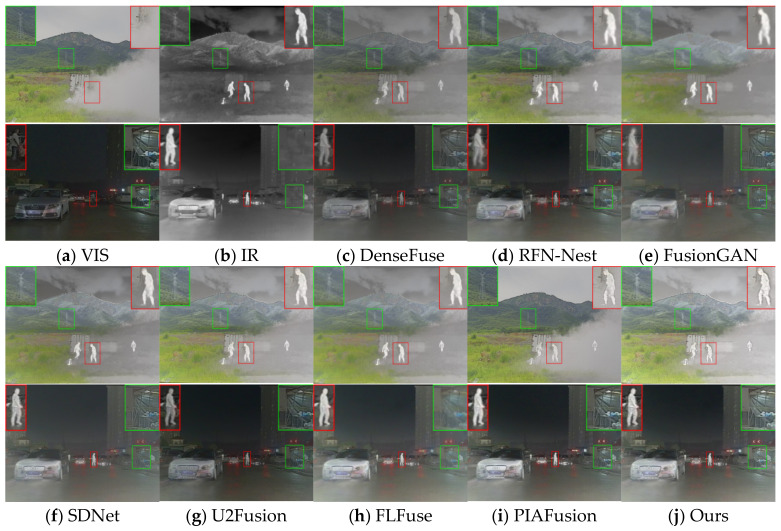
Vision quality comparison on the M3FD dataset. Areas with large differences are highlighted by RED and GREEN boxes, and enlarged images of RED boxes are in the lower right or left corner.

The visualization results for eight image pairs in the three datasets are given in Figure 5, Figure 6 and Figure 7.

In the daytime scene, as shown in Figure 5, DenseFuse and RFN-Nest weaken the infrared target, and FusionGAN causes the problem of blurred edge texture, while SDNet and FLFuse weaken the background texture detail of the image, as seen in the green box; only PIAFusion and the method in this paper can integrate the effective information.

In the night scene as shown in Figure 6, the visible image contains only a small amount of texture information, while the infrared image has background texture detail information in addition to the prominent target. Many methods focus excessively on the information of one of the modal images, and it is difficult to achieve good results in different scenes. Among them, the infrared targets in DenseFuse, RFN-Nest, U2Fusion and FLFuse are weakened, and the fused images of FusionGAN and SDNet are more towards the infrared images, resulting in blurred background information. Since PIAFusion adds light perception coefficients to the loss function, the method in this paper fully extracts the details contained in both images in the texture mainstream and uses saliency tributaries to supplement the weakened salient targets, so it can effectively fuse the complementary information in low-light scenes.

In the scenes where the visible image targets are obscured as shown in Figure 7, the method in this paper can mine the salient targets hidden in smoke because the method in this paper uses intermediate salient loss to guide the tributaries to enhance their strong contrast. Among the seven comparison algorithms, DenseFuse can retain texture information but ignores the salient contrast of the target, while background information is smoothed to different levels in RFN-Nest, FusionGAN, SDNet, U2Fusion, and FLFuse. In contrast, although PIAFusion can better preserve the high contrast of salient targets, it is easy to lose the IR modal information of obscured objects such as sky and smoke due to the smoothness of light perception loss.

In summary, our method has both comprehensive scene information and retains rich contrast information and texture details of the target region.

#### 4.2.2. Quantitative Results

We performed a quantitative evaluation on three datasets, TNO, MARS, and M3FD.The comparison of the metrics of different methods is shown in Table 1 below. The best values of AG and SF indicate that our fusion method has richer contrast information and also contains richer texture details; the best value of EN indicates that our method retains sufficient edge information; and the best value of SCD indicates that our fusion results contain more realistic information. SD and VIF perform optimal or suboptimal on the three datasets, indicating that our method has richer contrast information and generates fused images that are more consistent with the human visual system. In addition, six metrics are optimal or suboptimal on three datasets indicating that our method has superior generalization performance and can be applied to different types of datasets. In conclusion, our method is able to mine effective information in low-light and occluded scenes and integrate the information into the fused images with the help of spatially paranoid blocks. Therefore, our method has a greater advantage over other methods to obtain high-quality fused images.

### 4.3. Application of Semantic Segmentation

**Figure 8:**
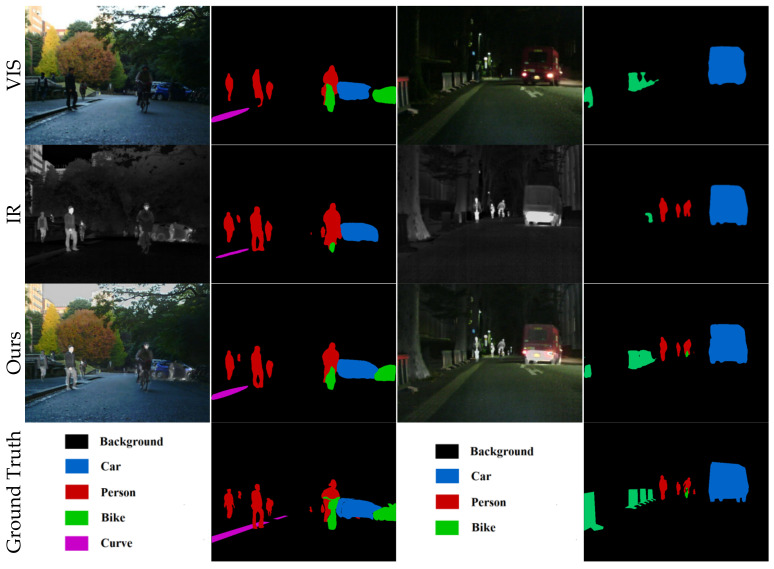
Vision quality comparison of the segmentation results.

In this section we validate the facilitation of this paper’s approach for advanced vision on a semantic segmentation task [29]. Specifically, we train the semantic segmentation algorithm [42] on the source and fused images, respectively. We selected 1000 images as the training set and tested the segmentation performance of different models on 360 images, and the qualitative and quantitative results are shown in Figure 8 and Table 2.

In the daytime scene as shown in columns one and two of Figure 8, the visible images contain a large amount of information, so the segmentation accuracy for visible images is high as shown in the second row of Table 2. However, some detection of people is lost due to the lack of guidance of infrared targets in the visible image. Additionally, the infrared image lacks the complement of the visible image background, and the segmentation accuracy of the bicycle is low as shown in the sixth column of the third row of Table 2.

In the night scene, as shown in Figure 8, columns three and four, the visible image cannot capture enough information due to the lack of light, so the segmentation network has a low segmentation accuracy for people in the scene, as shown in Table 2, fifth row, fifth column. While the infrared image captures the thermal target so the segmentation accuracy for people is higher as shown in the fifth column of the sixth row of Table 2; however, the infrared image reduces the segmentation accuracy of the bicycle.

Our method is shown in row three of Figure 8. Since the inclusion of the spatial bias term enables the network to perceive long-distance information and enhances the semantic information of the images, our method fully integrates the useful information of both source images, so our method outperforms the segmentation accuracy of pedestrians and bicycles than unimodal images in both daytime and nighttime scenes.

### 4.4. Ablation Experiment

**Figure 9:**
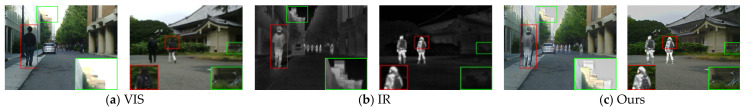

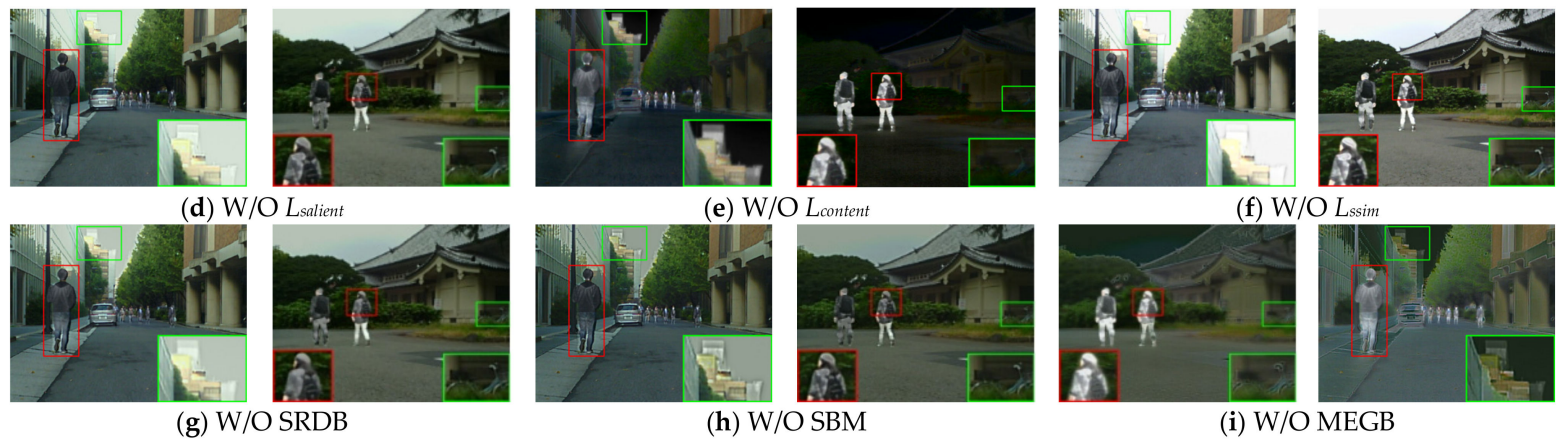
Vision quality comparison of the ablation study on important loss functions and modules.

In this section, we qualitatively and quantitatively analyze the effectiveness of the loss functions and modules in the method of this paper through ablation studies. The results are shown in Table 3 and Figure 9.

#### 4.4.1. Loss of Salience

The salient loss guides the tributary network to retain the high contrast of the infrared targets, aiming to compensate for the salient target features towards the feature mainstream. As shown in Figure 9d, the contrast of the targets marked in the red boxes significantly decreases after removing the salient loss, and the SD values (evaluated contrast) in Table 3 decrease, indicating that there is no salient loss, and the network’s infrared targets are weakened.

#### 4.4.2. Loss of Content 

Content loss uses intensity loss and gradient loss jointly to constrain the network to maintain the optimal intensity distribution while retaining abundant texture detail. As shown in Figure 9e, after removing the content loss, it is obvious that a significant decrease in background texture detail and a significant decrease in various metrics can be seen in the fused image biased toward the infrared image, which shows that the content loss has an important role in the overall network to synthesize the characteristics of the infrared and visible images.

#### 4.4.3. Structural Similarity Loss

The structural similarity loss aims to measure the similarity of the fused image to the source image. As shown in Figure 9f, when the structural similarity loss is removed, over-exposure is perpetuated in the visible image overexposure region for the fused image. On the other hand, the values of SD and EN vary greatly, indicating that the fused image contains less information with lower image contrast.

#### 4.4.4. Salient Dense Residual Block

SRDB utilizes attention to enable network features to extract a strong pixel distribution in the attention channel. As shown in Figure 9g, after removing the saliency-dense residual blocks, we can notice a significant decrease in the saliency of the fused image targets. The value of SD in Table 3 significantly decreases, indicating that the attention block is critical to the strong pixel distribution.

#### 4.4.5. Spatial Bias Block

The SBM effectively completes the progressive fusion process by adding information from the salient tributaries to the main stream. In Figure 9h and Table 3, it can be seen that the overall brightness of the fused image becomes darker and the target contrast decreases after removing the spatial bias block (SBM). On the other hand, the values of VIF, SCD and SD decrease significantly, which shows that adding spatial bias terms to the tributary can both effectively enhance the IR target and fused image more in line with the human visual system.

#### 4.4.6. Multiscale Edge Gradient Block

MEGB can fully extract the texture information of the image by using multiscale feature extraction with gradient operator embedding. As shown in Figure 9i, when we exclude the multiscale edge gradient block, the overall scene is relatively smoother with less gradient variation. Additionally, the values of AG and SF in Table 3 drop significantly, indicating that the module does enhance the representation of network texture details.

In summary, our designed module not only facilitates the fusion image visually, but also improves significantly in terms of metrics, so our designed module facilitates the maintenance of both texture and salient targets.

## 5. Summary

This paper proposed a saliency-compensated infrared and visible image fusion method, SCFusion. On the one hand, MEGB helps the extraction and retention of texture gradients of the overall network, which enhances the ability of the fused image to describe the global scene information. On the other hand, SRDB is designed to extract salient targets of infrared images and generate salient maps guided by salient loss. Finally, the information fusion is completed by compensating the saliency features of the tributaries into the main stream using SBM blocks. The experiments comparing the qualitative and quantitative aspects of this paper’s method with existing methods show the effectiveness of this paper’s method, and the fusion experiments with different lighting scenes also show that this paper’s method can effectively help to fully fuse the information of infrared and visible images in low-light scenes. Moreover, experiments on our semantic segmentation task validate the facilitation of our approach for subsequent high-level vision tasks. However, there are limitations to our method. Although our method can mitigate the loss of fused image scene information when the visible image is obscured by smoke to some extent, our method cannot remove the overexposure effect caused by strong light interference. We will further investigate the combination of low-light enhancement and image fusion tasks to solve the problem of strong light interference in the future.

## Figures and Tables

**Table 1:** Quantitative results of six metrics on TNO, MSRS and M3FD datasets. Bold: best. Underline and italic: second best.

Dataset	Algorithm	Evaluation Methods					
		SD	VIF	AG	SCD	EN	SF
TNO	DenseFuse	8.5765	0.6704	2.4895	1.5916	6.3422	0.0248
	RFN-Nest	*9.3153*	0.8103	2.6109	*1.7711*	*6.9285*	0.0226
	FusionGAN	8.6058	0.6457	2.3636	1.3688	6.5199	0.0240
	SDNet	9.0398	0.7523	*4.5252*	1.5488	6.6670	*0.0448*
	U2Fusion	8.8553	0.6787	3.4891	1.5862	6.4230	0.0327
	FLFuse	9.2156	0.7986	3.2772	1.7172	6.6307	0.0329
	PIAFusion	9.1093	**0.8835**	4.4265	1.6540	6.8937	0.0447
	Ours	**9.7039**	*0.8121*	**5.5097**	**1.8117**	**7.0620**	**0.0502**
MSRS	DenseFuse	7.0692	0.6752	2.0412	1.3296	5.8397	0.0235
	RFN-Nest	6.9939	0.5364	1.5376	1.2881	5.7514	0.0181
	FusionGAN	5.4307	0.4234	1.2258	0.7948	5.2179	0.0146
	SDNet	5.3143	0.3745	2.1439	0.8298	4.8852	0.0270
	U2Fusion	5.6231	0.3967	2.0100	1.0034	4.7525	0.0256
	FLFuse	6.4837	0.4837	1.7743	1.1090	5.5079	0.0193
	PIAFusion	**7.9268**	**0.9072**	**3.6801**	*1.7395*	**6.4304**	**0.0444**
	Ours	*7.7783*	*0.7354*	*3.3791*	**1.8057**	*6.4044*	*0.0421*
M3FD	DenseFuse	8.6130	0.6694	2.6528	1.5051	6.4264	0.0298
	RFN-Nest	9.0712	0.7338	2.5848	*1.6352*	6.7151	0.0274
	FusionGAN	8.8489	0.5154	2.3610	1.1257	6.4690	0.0274
	SDNet	8.8867	0.6321	4.0228	1.3912	6.6134	0.0454
	U2Fusion	9.0141	0.7061	3.8500	1.5488	6.6285	0.0408
	FLFuse	8.7556	0.6969	2.1329	1.4934	6.5734	0.0233
	PIAFusion	**10.1639**	**0.9300**	*4.9702*	1.3363	*6.8036*	*0.0575*
	Ours	*9.4840*	*0.7894*	**5.4374**	**1.7589**	**6.9482**	**0.0606**

**Table 2:** Segmentation performance (mIoU) of visible, infrared, and fused images at different times in the same scene. (Bold: best.).

Label Class		Background	Car	Person	Bike	Curve	Car Stop	Guardrail	Color Cone	Bump	Mean
Day	VIS	0.9800	0.8906	0.5556	0.7260	**0.5798**	0.4824	**0.8090**	**0.6508**	**0.5669**	0.6934
	IR	0.9482	0.5470	0.6564	0.0847	0.1032	0.1268	0.0368	0.0087	0.1304	0.2936
	Ours	**0.9834**	**0.9074**	**0.7332**	**0.7347**	0.5469	**0.5395**	0.7588	0.6335	0.5534	**0.7101**
Night	VIS	0.9652	0.6960	0.1305	0.5889	0.2750	0.1762	**0.3666**	0.3792	0.1943	0.4191
	IR	0.9593	0.4680	0.7103	0.0873	0.2599	0.0292	0.0000	0.0223	0.1945	0.3034
	Ours	**0.9763**	**0.7902**	**0.7205**	**0.6057**	**0.4419**	**0.2881**	0.3390	**0.4354**	**0.2233**	**0.5356**
All	VIS	0.9726	0.7933	0.3431	0.6575	0.4274	0.3293	**0.5878**	0.5150	0.3806	0.5563
	IR	0.9538	0.5075	0.6834	0.0860	0.1816	0.0780	0.0184	0.0155	0.1625	0.2985
	Ours	**0.9799**	**0.8488**	**0.7269**	**0.6702**	**0.4944**	**0.4138**	0.5489	**0.5345**	**0.3884**	**0.6229**

**Table 3:** Quantitative evaluation results of ablation study. (Bold: best).

Experiment	Evaluation Methods					
	SD	VIF	AG	SCD	EN	SF
Ls + SRDB + SBM + MEGB	7.7783	0.7354	**3.3791**	**1.8057**	**6.4044**	**0.0421**
W/O L_Salient_	7.7368	**0.7765**	3.2551	1.6536	6.1237	0.0420
W/O L_Content_	5.9613	0.6719	1.9179	0.9957	5.4447	0.0239
W/O L_SSIM_	6.9871	0.6764	3.1844	1.4672	5.9284	0.0400
W/O SRDB	7.6180	0.7489	3.3681	1.5740	6.1327	0.0414
W/O SBM	7.4222	0.5957	3.3583	1.2744	5.9838	0.0403
W/O MEGB	**7.8551**	0.4782	3.1330	0.7154	6.0242	0.0385

## Data Availability

Not applicable.
